# Subtrochanteric Fracture following Removal of a Porous Tantalum Implant

**DOI:** 10.1155/2013/946745

**Published:** 2013-12-03

**Authors:** Derek F. Amanatullah, Randall Farac, Thomas J. McDonald, H. David Moehring, Paul E. Di Cesare

**Affiliations:** ^1^Mayo Clinic, Department of Orthopaedic Surgery, 200 First Street SW, Gonda 14, Rochester, MN 55905, USA; ^2^Pacific Orthopaedic Associates, 707 South Garfield Avenue, 2nd Floor, Alhambra, CA 91801, USA; ^3^Sierra Orthopaedic Institute, 860 Guzzi Lane, Suite 105, Sonora, CA 95370, USA; ^4^University of California Davis Medical Center, Department of Orthopaedic Surgery, 4860 Y Street, Suite 3800, Sacramento, CA 95817, USA; ^5^New York Hospital Queens, 56-45 Main Street, 4th Floor South, Flushing, NY 11355, USA

## Abstract

Osteonecrosis of the hip accounts for about 10% of all total hip arthroplasty cases and presents a significant challenge for those patients with and without femoral head collapse. Subtrochanteric femur fractures have been reported with numerous types of proximal femoral implants. Care must be taken to avoid penetrating the lateral cortex of the proximal femur inferior to the distal border of the lesser trochanter. Core decompression requires a 3 mm to 20 mm defect in the lateral femoral cortex. Subtrochanteric femur fractures are a well-known complication of core decompression as well. We present a case of a subtrochanteric fracture following the removal of a porous tantalum implant.

## 1. Introduction

Osteonecrosis of the hip accounts for 8% to 12% of all total hip arthroplasty cases and presents a significant challenge for patients with and without femoral head collapse [1]. The current diagnostic and surgical approaches for avascular necrosis of the femoral head were recently reviewed in [2, 3].

Core decompression was developed by Ficat and Arlet during their acquisition of biopsy specimens. Their procedure anecdotally resulted in decreased hip pain. Most proponents of core decompression believe it is beneficial for Ficat stage I and II, especially compared with activity modification alone [4–10]. Some investigators have proposed supplementing core decompression with a non-vascularized or vascularized bone graft to improve subchondral support and enhance femoral head remodeling. Core decompression alone, with nonvascularized bone graft, or with a vascularized free fibula graft have widely variable results [4–15]. In addition, vascularized fibula harvest may result in donor site morbidity.

Porous tantalum rod implantation was designed to provide structural support to the femoral head and subtrochanteric femur after core decompression. Retrospective evaluation of 60 hips with osteonecrosis of the femoral head before radiographic femoral head collapse managed with core decompression and a porous tantalum rod demonstrated 92% at 1-year, 82% at 2-year, and 68% at 4-year implant survival [16]. Another retrospective study of 26 hips with osteonecrosis of the femoral head at various stages of femoral head collapse (Ficat II to IV) supports this observation reporting 70% survival at 6 years [17]. However, 50% of these cases demonstrated continued femoral head collapse [17]. Conversion to total hip arthroplasty was more common with more advanced disease [17]. Other retrospective studies of hips with advanced Ficat stages corroborate a mean time to failure of less than 1 year [18]. Closer scrutiny demonstrates a 14% rate of conversion to total hip arthroplasty with porous tantalum rods and a 33% rate of conversion to total hip arthroplasty with core decompression and vascularized free fibula transfer at 3 years [19].

## 2. Case Report

**Figure 1:**
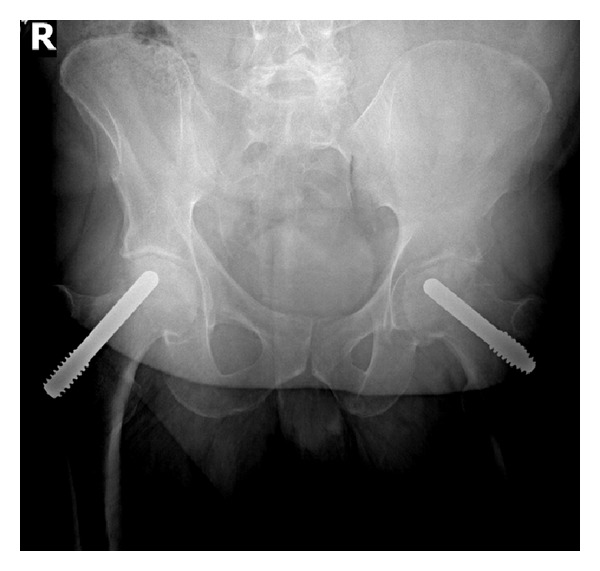
Preoperative pelvis radiograph showing bilateral porous tantalum implants.

A 36-year-old male presented with the chief complaint of severe bilateral hip pain. He had previously been diagnosed with avascular necrosis of the femoral head. The etiology of the avascular necrosis was unknown. Approximately one year prior, he had undergone placement of bilateral Osteonecrosis Intervention Implant (Trabecular Metal; Zimmer Trabecular Metal Technology, Allendale, NJ, USA). The patient reported a brief period of symptomatic improvement following this procedure, but his hip pain ultimately progressed resulting in marked functional impairment. His past medical history included type II diabetes mellitus and hypertension. The preoperative physical examination was significant for an antalgic gait as well as pain with passive internal and external hip rotation bilaterally. Radiographs at the initial visit showed Ficat stage III bilateral hip avascular necrosis with femoral head collapse and the presence of porous tantalum implants within each femoral neck and head (Figure 1). Serology was unremarkable and no hip aspiration was performed. A staged treatment plan was proposed consisting of Osteonecrosis Intervention Implant removal, intraoperative culture and sensitivity studies, and subsequent total hip arthroplasty once infection had been excluded.

**Figure 2:**
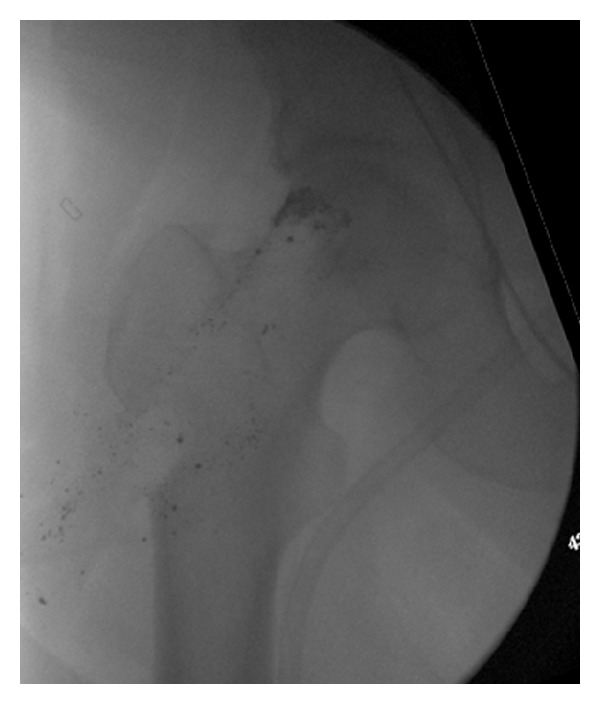
Postoperative radiograph of right proximal femur.

The patient was taken to the operating room; the left Osteonecrosis Intervention Implant was carefully overreamed by 0.5 mm using a hand trephine. Once the tip of the Osteonecrosis Intervention Implant was reached, the implant was manually backed out of the femoral head and neck using the manufacturer's extraction device. Specimens of surrounding tissue and bone were sent for culture. A similar approach was used for removing the contralateral rod. More difficulty was encountered on the right side due to a greater amount of host bone ingrowth. The Osteonecrosis Intervention Implant was over-reamed by 1 mm to extract the device. Despite the surgeons' effort to remove all metallic fragments and remove as little host bone as necessary, the postoperative radiographs did reveal retained metallic fragments in both hips as well as significant bone loss from the femoral metaphysis and neck (Figure 2).

**Figure 3:**
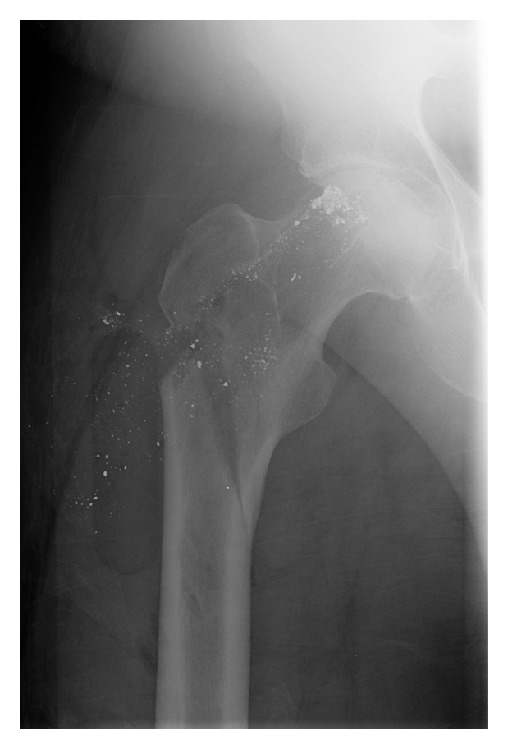
Postoperative day 1 right hip radiograph demonstrating a subtrochanteric femur fracture after the patient rolled in bed and heard a “crack.”

**Figure 4:**
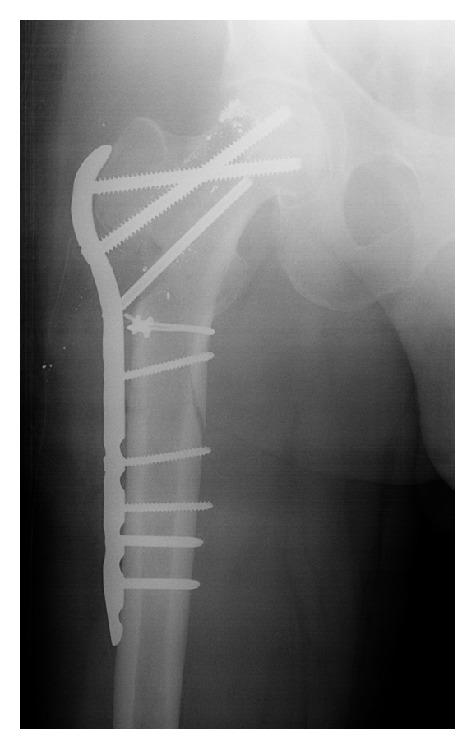
Right hip radiograph after open reduction and internal fixation.

Early the next morning, the patient noted severe pain in his right hip and proximal thigh associated with a loud “crack” when repositioning in bed. Radiographic evaluation of the right hip revealed an oblique fracture within the intertrochanteric and subtrochanteric regions of the proximal femur, originating from the defect left by the removed Osteonecrosis Intervention Implant (Figure 3). The fracture was managed with open reduction and internal fixation using a proximal femoral locking plate and went on to union (Figure 4). Arthroplasty was still considered a salvage option at this time because of the patient's age and concern for persistent occult infection. Intraoperative cultures were positive for *Propionibacterium acnes*. This infection was treated with 6 weeks of intravenous Ceftriaxone and 3 months of oral Doxycycline as recommended by the infectious disease consultant. No clinical signs of infection developed.

## 3. Discussion

Subtrochanteric femur fractures have been reported with numerous types of proximal femoral implants [20–23]. Care must be taken to avoid penetrating the lateral cortex of the proximal femur inferior to the distal border of the lesser trochanter [21, 22]. Core decompression requires a 3 mm to 20 mm defect in the lateral femoral cortex; hence subtrochanteric femur fractures are a well-known complication of core decompression. Finite element analysis suggests that larger defects increase the risk of subtrochanteric femur fractures. Finite element analysis predicts that multiple small drill holes are superior to one large drill hole created by the insertion of a porous tantalum implant or vascularized fibular graft during core decompression [24]. Retrospective analysis of 40 core decompressions revealed 3 (7.5%) subtrochanteric femur fractures [25]. Another retrospective study of 707 consecutive core decompressions with vascularized fibula grafts had 18 (2.5%) subtrochanteric femur fractures [20]. Seventeen of the 18 subtrochanteric femur fractures were due to falls or abnormal pivoting during the initial period of protected weight bearing. Subtrochanteric femur fractures were reported during the insertion, but not after the removal, of porous tantalum implants [26].

The use of a porous tantalum implant following a core decompression for the treatment of early-stage osteonecrosis of the femoral head is appealing as it is easy to insert and avoids the morbidity of harvesting the fibula for graft. However, this is a proposed mechanical solution for a fundamentally biologic problem. Retrieval studies of 15 porous tantalum implants demonstrate bone ingrowth in only 13 (87%) and continued subchondral collapse in 9 (60%) despite a porosity and an elastic modulus of the tantalum implants that mimic bone [27–29]. We elected to remove this implant because of progressive pain and to rule out occult infection prior to total hip arthroplasty. It is unclear if this is necessary or even desirable as the extraction of the porous tantalum implant after osseous integration resulted in a significant amount of retained metal, bone loss, and a pathological subtrochanteric femur fracture. One stage conversion of porous tantalum implants to total hip arthroplasty is reported [19]. It should be noted that porous tantalum implants may preclude hip resurfacing arthroplasty which might be selected for a young active patient.

## 4. Conclusion

Subtrochanteric femur fracture can occur during the insertion or removal of a porous tantalum rod for osteonecrosis of the femoral head. This implant creates a large lateral cortical defect in the femur that may be below the level of the lesser trochanter.

## References

[1] Mont MA, Hungerford DS (1995). Non-traumatic avascular necrosis of the femoral head. *Journal of Bone and Joint Surgery A*.

[2] Amanatullah DF, Strauss EJ (2011). Current management options for osteonecrosis of the femoral head: part II, operative management. *The American Journal of Orthopedics*.

[3] Amanatullah DF, Strauss EJ, Di Cesare PE (2011). Current management options for osteonecrosis of the femoral head: part 1, diagnosis and nonoperative management. *American Journal of Orthopedics*.

[4] Ficat RP (1985). Idiopathic bone necrosis of the femoral head. Early diagnosis and treatment. *Journal of Bone and Joint Surgery B*.

[5] Koo K-H, Kim R, Ko G-H, Song H-R, Jeong S-T, Cho S-H (1995). Preventing collapse in early osteonecrosis of the femoral head. A randomised clinical trial of core decompression. *Journal of Bone and Joint Surgery B*.

[6] Markel DC, Miskovsky C, Sculco TP, Pellicci PM, Salvati EA (1996). Core decompression for osteonecrosis of the femoral head. *Clinical Orthopaedics and Related Research*.

[7] Mont MA, Carbone JJ, Fairbank AC (1996). Core decompression versus nonoperative management for osteonecrosis of the hip. *Clinical Orthopaedics and Related Research*.

[8] Smith SW, Fehring TK, Griffin WL, Beaver WB (1995). Core decompression of the osteonecrotic femoral head. *Journal of Bone and Joint Surgery A*.

[9] Stulberg BN, Davis AW, Bauer TW, Levine M, Easley K (1991). Osteonecrosis of the femoral head: a prospective randomized treatment protocol. *Clinical Orthopaedics and Related Research*.

[10] Fairbank AC, Bhatia D, Jinnah RH, Hungerford DS (1995). Long-term results of core decompression for ischaemic necrosis of the femoral head. *Journal of Bone and Joint Surgery B*.

[11] Chen C-C, Lin C-L, Chen W-C, Shih H-N, Ueng SWN, Lee MS (2009). Vascularized iliac bone-grafting for osteonecrosis with segmental collapse of the femoral head. *Journal of Bone and Joint Surgery A*.

[12] Marciniak D, Furey C, Shaffer JW (2005). Osteonecrosis of the femoral head: a study of 101 hips treated with vascularized fibular grafting. *Journal of Bone and Joint Surgery A*.

[13] Roush TF, Olson SA, Pietrobon R, Braga L, Urbaniak JR (2006). Influence of acetabular coverage on hip survival after free vascularized fibular grafting for femoral head osteonecrosis. *Journal of Bone and Joint Surgery A*.

[14] Urbaniak JR, Coogan PG, Gunneson EB, Nunley JA (1995). Treatment of osteonecrosis of the femoral head with free vascularized fibular grafting: a long-term follow-up study of one hundred and three hips. *Journal of Bone and Joint Surgery A*.

[15] Zhao D, Xu D, Wang W, Cui X (2006). Iliac graft vascularization for femoral head osteonecrosis. *Clinical Orthopaedics and Related Research*.

[16] Veillette CJH, Mehdian H, Schemitsch EH, Mckee MD (2006). Survivorship analysis and radiographic outcome following tantalum rod insertion for osteonecrosis of the femoral head. *Journal of Bone and Joint Surgery A*.

[17] Varitimidis SE, Dimitroulias AP, Karachalios TS, Dailiana ZH, Malizos KN (2009). Outcome after tantalum rod implantation for treatment of femoral head osteonecrosis: 26 hips followed for an average of 3 years. *Acta orthopaedica*.

[18] Nadeau M, Séguin C, Theodoropoulos JS, Harvey EJ (2007). Short term clinical outcome of a porous tantalum implant for the treatment of advanced osteonecrosis of the femoral head. *McGill Journal of Medicine*.

[19] Shuler MS, Rooks MD, Roberson JR (2007). Porous tantalum implant in early osteonecrosis of the hip. Preliminary report on operative, survival, and outcomes results. *Journal of Arthroplasty*.

[20] Aluisio FV, Urbaniak JR (1998). Proximal femur fractures after free vascularized fibular grafting to the hip. *Clinical Orthopaedics and Related Research*.

[21] DiMaio FR, Haher TR, Splain SH, Mani VJ (1992). Stress-riser fractures of the hip after sliding screw plate fixation. *Orthopaedic Review*.

[22] Kloen P, Rubel IF, Lyden JP, Helfet DL (2003). Subtrochanteric fracture after cannulated screw fixation of femoral neck fractures: a report of four cases. *Journal of Orthopaedic Trauma*.

[23] Toriumi H, Miyasaka T, Uchiyama S, Nakagawa H (1998). Utilization of a partially threaded kirschner wire in the treatment of femoral neck fractures. *Journal of Orthopaedic Trauma*.

[24] Floerkemeier T, Lutz A, Nackenhorst U (2011). Core decompression and osteonecrosis intervention rod in osteonecrosis of the femoral head: clinical outcome and finite element analysis. *International Orthopaedics*.

[25] Camp JF, Colwell CW (1986). Core decompression of the femoral head for osteonecrosis. *Journal of Bone and Joint Surgery A*.

[26] Stronach BM, Duke JN, Rozensweig SD, Stewart RL (2010). Subtrochanteric femur fracture after core decompression and placement of a tantalum strut for osteonecrosis of the femoral head. *Journal of Arthroplasty*.

[27] Tanzer M, Bobyn JD, Krygier JJ, Karabasz D (2008). Histopathologic retrieval analysis of clinically failed porous tantalum osteonecrosis implants. *Journal of Bone and Joint Surgery A*.

[28] Bobyn JD, Poggie RA, Krygier JJ (2004). Clinical validation of a structural porous tantalum biomaterial for adult reconstruction. *Journal of Bone and Joint Surgery A*.

[29] Tsao AK, Roberson JR, Christie MJ (2005). Biomechanical and clinical evaluations of a porous tantalum implant for the treatment of early-stage osteonecrosis. *Journal of Bone and Joint Surgery A*.

